# Peripheral NMDA Receptors Mediate Antidromic Nerve Stimulation-Induced Tactile Hypersensitivity in the Rat

**DOI:** 10.1155/2015/793624

**Published:** 2015-12-03

**Authors:** Jun Ho Jang, Taick Sang Nam, Jaebeom Jun, Se Jung Jung, Dong-Wook Kim, Joong Woo Leem

**Affiliations:** ^1^Dental Research Institute, Seoul National University, Seoul 110-744, Republic of Korea; ^2^Department of Physiology, Yonsei University College of Medicine, Seoul 120-752, Republic of Korea; ^3^Brain Research Institute, Yonsei University College of Medicine, Seoul 120-752, Republic of Korea; ^4^Brain Korea 21 PLUS Project for Medical Science, Yonsei University College of Medicine, Seoul 120-752, Republic of Korea; ^5^Graduate Program in Cognitive Science Yonsei University, Seoul 120-752, Republic of Korea

## Abstract

We investigated the role of peripheral NMDA receptors (NMDARs) in antidromic nerve stimulation-induced tactile hypersensitivity outside the skin area innervated by stimulated nerve. Tetanic electrical stimulation (ES) of the decentralized L5 spinal nerve, which induced enlargement of plasma extravasation, resulted in tactile hypersensitivity in the L4 plantar dermatome of the hind-paw. When intraplantar (i.pl.) injection was administered into the L4 dermatome before ES, NMDAR and group-I metabotropic Glu receptor (mGluR) antagonists and group-II mGluR agonist but not AMPA/kainate receptor antagonist prevented ES-induced hypersensitivity. I.pl. injection of PKA or PKC inhibitors also prevented ES-induced hypersensitivity. When the same injections were administered after establishment of ES-induced hypersensitivity, hypersensitivity was partially reduced by NMDAR antagonist only. In naïve animals, i.pl. Glu injection into the L4 dermatome induced tactile hypersensitivity, which was blocked by NMDAR antagonist and PKA and PKC inhibitors. These results suggest that the peripheral release of Glu, induced by antidromic nerve stimulation, leads to the expansion of tactile hypersensitive skin probably via nociceptor sensitization spread due to the diffusion of Glu into the skin near the release site. In addition, intracellular PKA- and PKC-dependent mechanisms mediated mainly by NMDAR activation are involved in Glu-induced nociceptor sensitization and subsequent hypersensitivity.

## 1. Introduction

Primary afferent nociceptors are responsible for converting harmful stimuli in the peripheral tissue into internal electrical impulses and then conveying these impulses to the central nervous system for pain perception. In addition to their afferent functions, evidence indicates that nociceptors also have local efferent functions. Upon activation, nociceptive afferents, especially the unmyelinated C-fibers that constitute the largest proportion of cutaneous nociceptive afferents, are able to provoke the local release of various algesic substances at the peripheral terminals [1–3]. Indeed, stimulation of C-fibers results in a local increase in levels of glutamate (Glu), substance P (SP), and calcitonin gene-related peptide (CGRP) [4–7]. Peripherally released algesic substances are known to cause neurogenic inflammation [1, 8] and are also likely to diffuse to receptors expressed on nearby nociceptors in a paracrine manner to modulate neuronal excitability.

There is significant evidence for the modulatory role of Glu in peripheral nociception. Both ionotropic and metabotropic Glu receptors (mGluRs) are present in the peripheral terminals of unmyelinated afferents [9–13]. The subcutaneous injection of Glu and specific GluR agonists produces pain behaviors that are blocked by their corresponding antagonists [10, 14]. Furthermore, local injection of GluR antagonists attenuates pain behaviors in various experimental models of pain [9, 15–24]. In addition, peripheral Glu is able to increase the excitability of nociceptors under both normal and pathological conditions [21, 25]. Glu-induced tactile hyperalgesia is mediated by intracellular signaling pathways that activate protein kinases such as protein kinases A (PKA) and C (PKC) in the primary sensory neurons [26, 27].

In a previous study using rats with an injury to the lumbar L5 spinal nerve (L5 SN) that had previously undergone a L5 dorsal root rhizotomy (DR) (decentralized L5 SN), we proposed Wallerian degeneration and peripherally propagating injury discharge induced by nerve ligation/cut as peripheral contributions to nerve injury-induced neuropathic pain [28]. The present study was performed using the same rhizotomized rats to investigate the role of peripherally propagating nerve impulses, which were evoked by a tetanic electrical stimulation (ES) that was revealed to release Glu from peripheral terminals of primary afferent fibers [4], in the development of hypersensitivity outside the skin area innervated by the stimulated nerve. To achieve this goal, we examined whether antidromic stimulation of the decentralized L5 SN induces tactile hypersensitivity on the L4 plantar dermatome of the hind-paw. In addition, we explored which subtypes of GluRs mediate such hypersensitivity. We also examined whether PKA and PKC were involved in this hypersensitivity.

## 2. Materials and Methods

### 2.1. Experimental Animals and Surgical Procedures

Adult male rats (150–250 g; Harlan Sprague-Dawley, Koatech Co., Gyeonggi-do, Korea) were used. The animals were housed in groups of three to four with food and water available* ad libitum*. All animals were acclimated to a 12-12 light-dark cycle for approximately 1 week before the surgery and behavioral testing. All the experiments were conducted in accordance with the approval of the Institutional Animal Care and Use Committee of Yonsei University, Seoul, Korea.

All the surgical procedures were conducted under enflurane anesthesia (2-3% enflurane-O_2_ mixture). The animals were initially subjected to an L5 DR before receiving subsequent manipulations. For the L5 DR, a longitudinal skin incision was made to expose the L3–L6 vertebral segments, and a hemilaminectomy was performed at the left L5 segments. Care was taken to avoid any mechanical trauma to the spinal cord, the dorsal roots, and the dorsal root ganglion (DRG). The dura mater was opened, and the left L5 dorsal root was exposed and sectioned 2-3 mm proximal to the L5 DRG. A small portion of the distal end of the dissected root was sectioned for removal.

### 2.2. Electrical Stimulation

**Figure 1:**
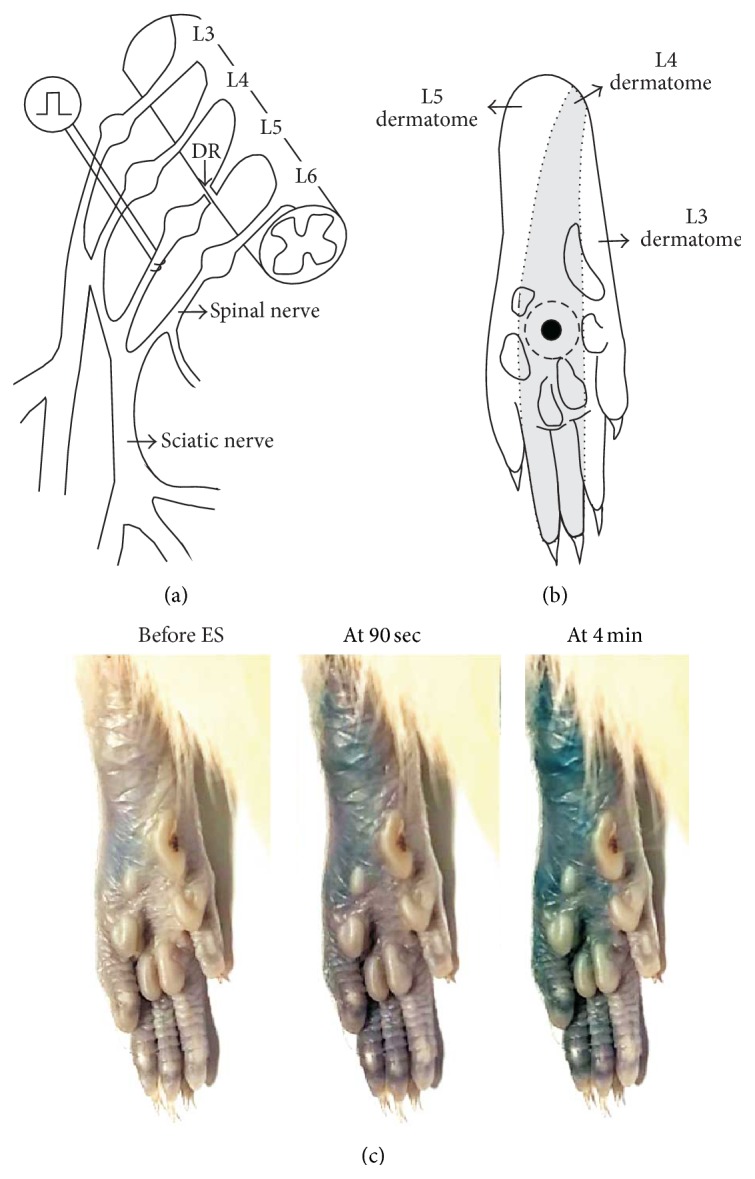
The locations of electrical stimulation (ES), intraplantar (i.pl.) injection of substances, and paw sensitivity testing and an example of ES-induced plasma extravasation. (a) An L5 spinal nerve (SN) was decentralized by L5 dorsal rhizotomy (DR). A high-level tetanic ES (2–4 mA, 0.5 ms pulse, 4 Hz, and 5 min) was applied to the decentralized L5 SN 4–6 mm distal to the L5 dorsal root ganglion (DRG). (b) The tactile sensitivity testing was performed in an area (marked by a filled circle) located in the center of the circle surrounded by the tori, which is almost matched to the midpoint of the L4 plantar dermatome (shaded area) [29] of the hind-paw. A 30 *μ*L i.pl. injection of different substances was administered subcutaneously into the skin area subjected to tactile sensitivity testing, forming a bleb that disappeared within 10 min after the injection (bleb area marked by a dotted circle). (c) Following Evans blue injection (i.v., 30 mg/kg, and 2% solution), areas of plasma extravasation induced by the ES of the L5 SN are seen as blue. Note that dye extravasation covered the L5 plantar dermatome within the first 90 seconds of the ES, and the extravasation area extended to the L4 plantar dermatome within 4 minutes of the ES.

A tetanic ES of the L5 SN was conducted in rats that received a left L5 DR (Figure 1(a)). In these rats, if mild tactile hyperalgesia had been present, then it had worn off within a week after the rhizotomy. The animals were under enflurane anesthesia throughout the ES experiment. After removing the left transverse process of the L6 vertebra, the left L5 SN was exposed. A piece of parafilm was placed underneath the exposed SN to isolate it from the surrounding tissue. A pair of thin flexible silver wires (0.005 in diameter) was placed 4–6 mm distal to the L5 DRG to gently loop around the L5 SN (3 mm apart). The other ends of the wires were connected to a stimulus isolation unit (SI-1850, World Precision Instruments, New Haven, CT, USA). Square-wave pulses (0.5 ms, 4 Hz) were applied with graded levels of current to determine the lowest level of current needed to elicit a muscle twitch or the threshold current. The current pulses at strengths 200 times the threshold current (2–4 mA, 200 × TH) were delivered for 5 min to activate both the A- and C-fibers in the L5 SN. For the sham-operated group, all procedures were conducted in exactly the same way as for the ES-treated animals, except that no ES was delivered.

The plasma extravasation, which is one of the characteristic symptoms of neurogenic inflammation, was induced by the ES of the L5 SN using extra animals that received an intravenous injection of Evans blue dye (Sigma, St. Louis, MO, USA) in the lateral tail vein (30 mg/kg, 2% solution) before the ES. The images of dye extravasation seen as blue on the plantar surface were captured with a digital video camera to determine whether changes in the extravasation area occurred during the ES.

### 2.3. Drug Preparation and Administration

Noncompetitive NMDA (*N*-methyl-d-aspartate) receptor (NMDAR) antagonist MK-801 and competitive AMPA (amino-3-hydroxy-5-methylisoxazole-4-propionic acid)/kainate receptor antagonist NBQX (2,3-dioxo-6-nitro-1,2,3,4,-tetrahydrobenzo[f]quinoxaline-7-sulfonamide) were dissolved in phosphate-buffered saline (PBS), pH 7.4. A competitive group-I mGluR antagonist DL-AP3 (DL-amino-3-phosphonopropionic acid) and selective group-II mGluR agonist APDC ((2R,4R)-4-aminopyrrolidine-2,4-dicarboxylate) were first prepared as a stock solution in 100 mM NaOH and then diluted to final concentrations with PBS. For PKA and PKC inhibition, staurosporine (a broad-spectrum kinase inhibitor) and calphostin C (a selective PKC inhibitor) were first prepared as a stock solution in 1 mM DMSO and then diluted to the final concentration with PBS. The Glu and H-89 (a selective PKA inhibitor) were dissolved in PBS. All the drugs were purchased from Tocris Cookson (Bristol, UK). Corresponding vehicles were prepared in an identical manner without the addition of the drug. The selection of drug doses used in this study was based on our previous study in which the effective doses of drugs without obvious side effects have been chosen [15]. These drug concentrations were lower than those used by other groups for various experiments, excluding the possibility of off-target effects [16–18, 30, 31].

Under enflurane anesthesia (3%, 2-3 min), 30 *μ*L of the drugs or corresponding vehicles was injected subcutaneously under the hind-paw skin using a 50 *μ*L Hamilton syringe (Reno, NV, USA) with a 28-gauge needle. For this intraplantar (i.pl.) injection, the needle was inserted into the skin in the middle of the plantar surface proximal to the proximal tori of the hind-paw and advanced approximately 10 mm so that it reached the center of the circle surrounded by the tori, where the solution was injected and formed a bleb that disappeared within 10 min (Figure 1(b)). Pretreatments were performed immediately (i.e., within 10 min) prior to the ES, and posttreatments were performed 3 days after the ES. To assess the antinociceptive effects of PKA and PKC inhibitors on Glu-induced hypersensitivity in naïve rats, pretreatment was conducted 10 min before the Glu treatment with both i.pl. injections administered at the same site.

### 2.4. Behavioral Testing

Each rat was placed in a plexiglass cage (8 × 8 × 20 cm^3^) above a wire mesh bottom that allowed full access to their paws. Following a 15 min acclimation period, the rats underwent behavioral testing. Tactile hypersensitivity was evaluated by measuring the paw withdrawal threshold (PWT) upon the application of a von Frey filament using the Dixon's up-down testing paradigm [32]. A series of von Frey filaments, ranging from 0.3 to 15.0 g, were applied perpendicular to the center of the circle surrounded by the tori of the hind-paw (avoiding the keratinized foot pads, see Figure 1(b)) for 2-3 s until each filament bent slightly, starting with a 2.5 g stimulus. For the PWT measurement, the 50% withdrawal threshold was determined based on an equation and the calibration table described by Chaplan et al. [33]. Behavioral testing was performed by investigators who were blind to the surgery and treatment that the animals had received.

### 2.5. Statistical Analysis

To determine the differences between the different treatment groups on a given testing day, the data were analyzed using a Mann-Whitney rank-sum test or a Kruskal-Wallis analysis of variance (ANOVA) followed by Dunn's test for multiple comparisons. The differences from baseline within a given treatment group were analyzed using a Friedman repeated measures ANOVA followed by Dunn's test for multiple comparisons. *P* < 0.05 was considered to be statistically significant. Data are represented as mean ± SEM.

## 3. Results

### 3.1. Tetanic ES-Induced Tactile Hypersensitivity

We investigated using rats with L5 DR whether a high-level tetanic ES (2–4 mA, 0.5 ms pulse, 4 Hz, and 5 min) of the L5 SN (L5 SN-ES) induced tactile hypersensitivity on the L4 dermatome. The tactile sensitivity was tested by measuring PWT using von Frey filaments applied to the center of the hind-paw glabrous skin surrounded by the tori, which is almost matched to the midpoint of the L4 plantar dermatome (Figure 1). This L5 SN-ES was observed to induce neurogenic inflammation consistently in all test animals, as judged by extravasated Evans blue, which covered the L5 plantar dermatome within the first 90 seconds of the ES and extended to the L4 plantar dermatome within 4 minutes of the ES. No further enlargement of the extravasation zone was observed during the remaining ES period. A typical example of ES-induced plasma extravasation observed in five rats tested was illustrated in Figure 1(c).

**Figure 2:**
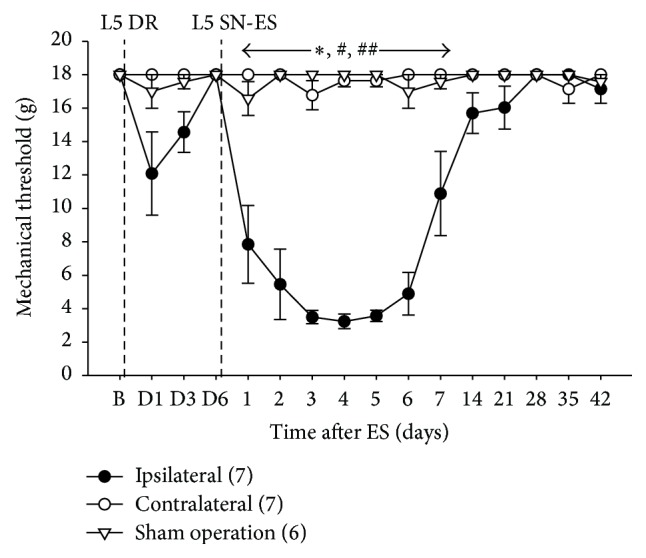
Tactile hypersensitivity following tetanic ES of the decentralized L5 SN. A tetanic ES of the L5 SN (L5 SN-ES) in rats with L5 DR induced a significant decrease in PWTs in the affected hind-paws on days 1 through 7 after ES compared with the baseline, contralateral hind-paws, and sham-operated animals. The abbreviations B and D denote the baseline before DR and postoperative days after DR, respectively. The figures in parentheses indicate the number of animals used. The error bars represent the SEM. ^*∗*^ *P* < 0.05 versus sham animals. ^#^ *P* < 0.05 versus baseline. ^##^ *P* < 0.05 versus contralateral hind-paws.

As for PWT changes (Figure 2), L5 SN-ES resulted in a significant decrease in the PWTs of the L4 plantar dermatome of the hind-paw ipsilateral to the ES as compared with the baseline. This decrease in the PWTs lasted for 7 days from poststimulation days 1 through 7. Such decreases also were significant in comparison with the PWTs of sham animals or the contralateral hind-paw.

### 3.2. Effects of Pretreatment in the Periphery with MK-801, NBQX, DL-AP3, or APDC on ES-Induced Tactile Hypersensitivity

**Figure 3:**
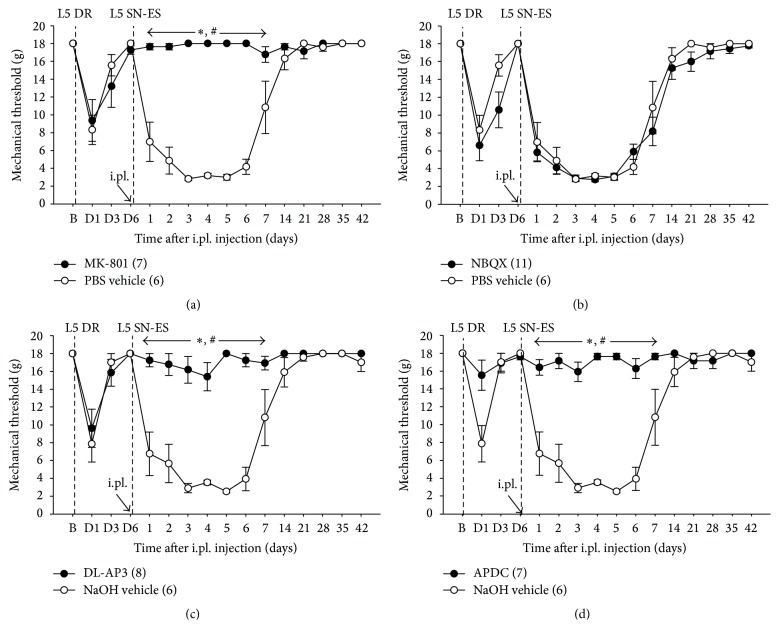
Effects of MK-801, NBQX, DL-AP3, or APDC pretreatment in the periphery on ES-induced PWT reduction. (a) In rats with L5 DR, an i.pl. injection of MK-801 (20 nM) administered immediately before the ES of L5 SN (L5 SN-ES) into the midpoint of the L4 plantar dermatome resulted in a significant increase in ES-induced decreased PWTs compared with vehicle-treated controls. (b) An i.pl. NBQX (100 nM) injection administered immediately prior to the ES produced no significant difference in ES-induced decreased PWTs from vehicle-treated controls. (c and d) Animals administered an i.pl. injection of DL-AP3 (70 nM) or APDC (20 nM) immediately prior to the ES exhibited a significant increase in ES-induced decreased PWTs compared with vehicle-treated controls. The abbreviations B and D are defined in Figure 2. The values in parentheses indicate the number of animals used. The error bars represent the SEM. ^*∗*^ *P* < 0.05 versus vehicle groups. ^#^ *P* < 0.05 versus baseline.

The involvement of NMDA and AMPA/kainate receptors in the initiation of L5 SN-ES-induced tactile hypersensitivity of the L4 plantar dermatome was examined using rats with decentralized L5 SN. As seen in Figure 3(a), an i.pl. MK-801 (20 nM) injection up to 10 min prior to the ES into the midpoint of the L4 plantar dermatome of the hind-paw ipsilateral to the ES resulted in a significant increase in ES-induced decreased PWTs compared with vehicle-treated controls. These data indicate that NMDARs in the L4 plantar dermatome contribute to the initiation of L5 SN-ES-induced tactile hypersensitivity. Animals administered an i.pl. NBQX (100 nM) injection prior to the ES exhibited no significant difference in ES-induced decreased PWTs compared with vehicle-treated controls (Figure 3(b)), which indicates that AMPA/kainate receptors in the L4 plantar dermatome are unlikely to contribute to the initiation of L5 SN-ES-induced hypersensitivity.

The involvement of mGluRs in the initiation of L5 SN-ES-induced tactile hypersensitivity of the L4 plantar dermatome was investigated. As seen in Figures 3(c) and 3(d), animals administered an i.pl. injection of a competitive group-I mGluR antagonist DL-AP3 (70 nM) or a selective group-II mGluR agonist APDC (20 nM) prior to the ES into the midpoint of the L4 plantar dermatome resulted in a significant increase in ES-induced decreased PWTs as compared with vehicle-treated controls. These data indicate that the activation of group-I mGluRs and the inhibition of group-II mGluRs in the L4 plantar dermatome are implicated in the initiation of L5 SN-ES-induced hypersensitivity.

### 3.3. Effects of Posttreatment in the Periphery with MK-801, NBQX, DL-AP3, or APDC on ES-Induced Tactile Hypersensitivity

**Figure 4:**
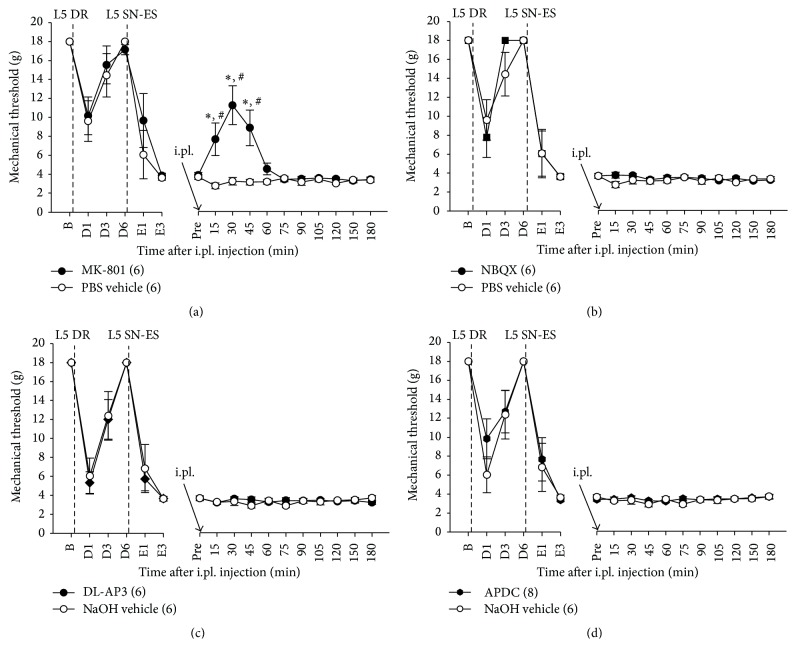
Effects of MK-801, NBQX, DL-AP3, or APDC posttreatment in the periphery on ES-induced PWT reduction. (a) In rats with L5 DR, an i.pl. injection of MK-801 (20 nM) administered 3 days after the ES of L5 SN (L5 SN-ES) into the midpoint of the L4 plantar dermatome resulted in a significant increase in ES-induced decreased PWTs at 15, 30, and 45 min after treatment compared with vehicle-treated controls. (b–d) No differences in ES-induced decreased PWTs were observed between animals treated with NBQX (100 nM), DL-AP3 (70 nM), or APDC (20 nM) and vehicle-treated controls. The abbreviations B, D, E, and Pre denote the baseline before DR, postoperative days after DR, postoperative days after ES, and baseline prior to i.pl. injection, respectively. The values in parentheses indicate the number of animals used. The error bars represent the SEM. ^*∗*^ *P* < 0.05 versus vehicle group. ^#^ *P* < 0.05 versus baseline.

The involvement of NMDA and AMPA/kainate receptors in the maintenance of L5 SN-ES-induced tactile hypersensitivity of the L4 plantar dermatome was examined using rats with a decentralized L5 SN. As seen in Figure 4(a), an i.pl. injection of MK-801 (20 nM) into the midpoint of the L4 plantar dermatome on day 3 after ES resulted in a significant increase in ES-induced decreased PWTs at 15, 30, and 45 min posttreatment compared with vehicle-treated controls, which indicates a contribution of NMDARs in the L4 plantar dermatome to the maintenance of L5 SN-ES-induced hypersensitivity. Animals administered an i.pl. injection of NBQX (100 nM) into the midpoint of the L4 plantar dermatome on day 3 after ES exhibited no significant changes in ES-induced decreased PWTs compared with vehicle-treated controls (Figure 4(b)), which indicates no participation of AMPA/kainate receptors in the L4 plantar dermatome in maintaining L5 SN-ES-induced hypersensitivity.

The involvement of group-I and -II mGluRs in the maintenance of L5 SN-ES-induced tactile hypersensitivity was tested. As seen in Figures 4(c) and 4(d), animals administered an i.pl. injection of DL-AP3 (70 nM) or APDC (20 nM) into the midpoint of the L4 plantar dermatome on day 3 after ES exhibited no significant changes in ES-induced decreased PWTs compared with vehicle-treated controls. These data indicate that neither group-I mGluRs nor group-II mGluRs in the L4 plantar dermatome contribute to the maintenance of L5 SN-ES-induced hypersensitivity.

### 3.4. Effects of Pre- and Posttreatment in the Periphery with Staurosporine, Calphostin C, or H-89 on ES-Induced Tactile Hypersensitivity

**Figure 5:**
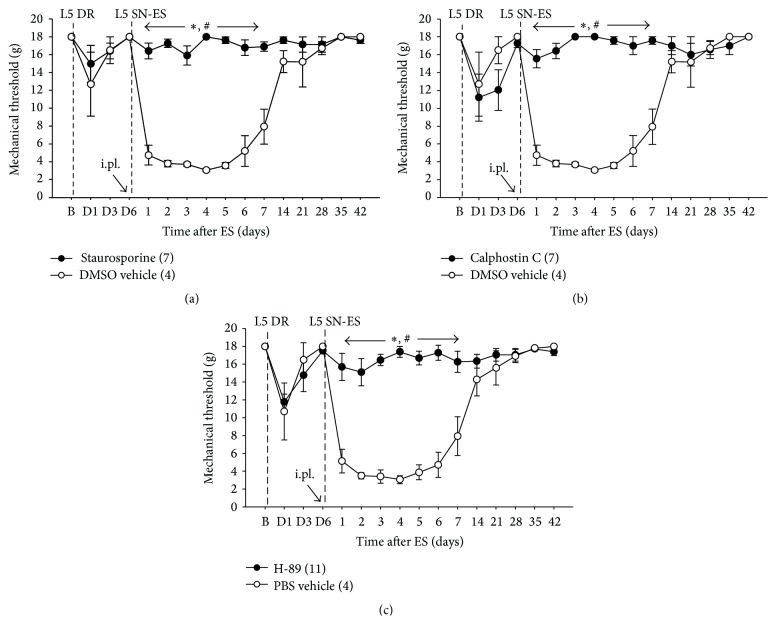
Effects of staurosporine, calphostin C, or H-89 pretreatment in the periphery on ES-induced PWT reduction. (a–c) In rats with L5 DR, an i.pl. injection of staurosporine (70 *μ*M), calphostin C (12 *μ*M), or H-89 (10 *μ*M) administered immediately prior to the ES of L5 SN (L5 SN-ES) into the midpoint of the L4 plantar dermatome resulted in a significant increase in ES-induced decreased PWTs compared with vehicle-treated controls. The abbreviations B and D are defined in Figure 2. The values in parentheses indicate the number of animals used. The error bars represent the SEM. ^*∗*^ *P* < 0.05 versus vehicle group. ^#^ *P* < 0.05 versus baseline.

The involvement of the intracellular signaling molecules PKA and PKC in the initiation of L5 SN-ES-induced tactile hypersensitivity was tested using rats with decentralized L5 SN. As seen in Figures 5(a), 5(b), and 5(c), an i.pl. injection of staurosporine (a broad-spectrum kinase inhibitor, 70 *μ*M), calphostin C (12 *μ*M), or H-89 (10 *μ*M) into the midpoint of the L4 plantar dermatome up to 10 min prior to the ES resulted in a significant increase in ES-induced decreased PWTs compared with vehicle-treated controls. These data indicate that both PKA and PKC participate in initiating L5 SN-ES-induced hypersensitivity in the L4 plantar dermatome.

**Figure 6:**
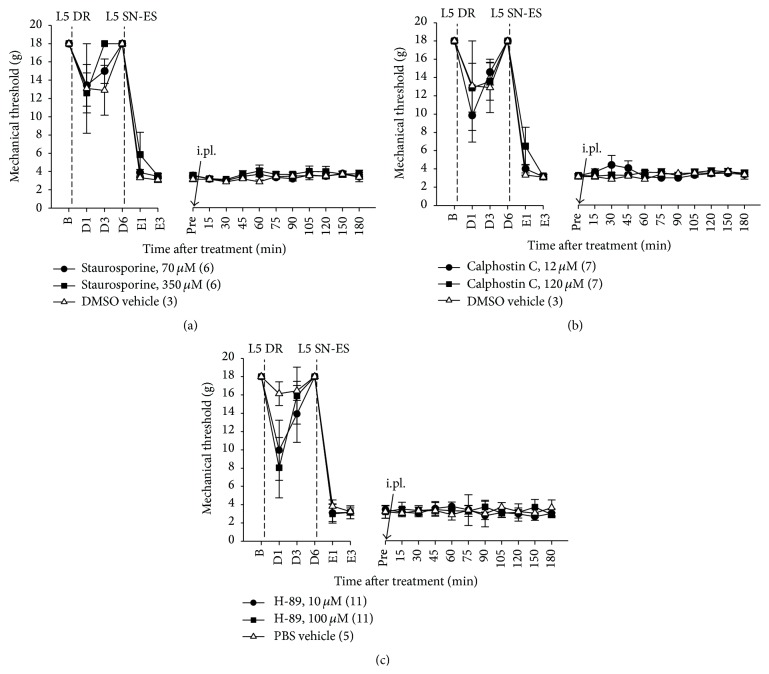
Effects of staurosporine, calphostin C, or H-89 posttreatment in the periphery on ES-induced PWT reduction. (a–c) In rats with L5 DR, an i.pl. injection of staurosporine (70 *μ*M), calphostin C (12 *μ*M), or H-89 (10 *μ*M) administered 3 days after the ES of L5 SN (L5 SN-ES) into the midpoint of the L4 plantar dermatome resulted in no significant difference in ES-induced decreased PWTs from vehicle-treated controls. No effects were observed with higher doses of each drug. The abbreviations B, D, E, and Pre are defined in Figure 4. The values in parentheses indicate the number of animals used. The error bars represent the SEM.

When an i.pl. injection was administered on day 3 after ES into the midpoint of the L5 plantar dermatome, animals treated with staurosporine (70 *μ*M), calphostin C (12 *μ*M), or H-89 (10 *μ*M) exhibited no significant changes in ES-induced decreased PWTs compared with vehicle-treated controls (Figures 6(a), 6(b), and 6(c)). No effects were observed at higher doses of each drug. Thus, neither PKA nor PKC are implicated in maintaining L5 SN-ES-induced hypersensitivity in the L4 plantar dermatome.

### 3.5. Effects of Pretreatment in the Periphery with MK-801, Calphostin C, or H-89 on Glu-Induced Tactile Hypersensitivity

**Figure 7:**
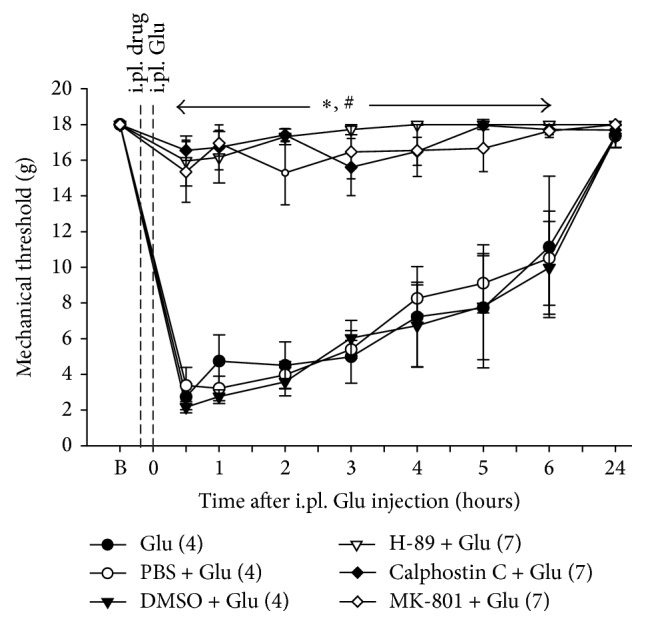
Effects of MK-801, calphostin C, or H-89 pretreatment in the periphery on Glu-induced PWT reduction. In naive rats, an i.pl. injection of Glu (1 *μ*M) into the midpoint of the L4 plantar dermatome resulted in a significant decrease in PWTs that lasted approximately 6 h compared with the baseline. Animals administered an i.pl. injection of MK-801 (20 nM), calphostin C (12 *μ*M), or H-89 (10 *μ*M) 10 min prior to the i.pl. injection of Glu at the same site exhibited a significant increase in Glu-induced decreased PWTs compared with vehicle-pretreated controls. B denotes the baseline before the i.pl. injections of the test substances. The values in parentheses indicate the number of animals used. The error bars represent the SEM. ^*∗*^ *P* < 0.05 versus vehicle group. ^#^ *P* < 0.05 versus baseline.

The involvement of NMDAR, PKA, and PKC in initiation of Glu-induced tactile hypersensitivity was investigated using naïve rats. As seen in Figure 7, an i.pl. injection of Glu into the midpoint of the L4 plantar dermatome of the hind-paw in naïve rats resulted in a significant decrease in PWTs, which lasted approximately 6 h compared with the baseline. An i.pl. injection of MK-801 (20 nM), calphostin C (12 *μ*M), or H-89 (10 *μ*M) into the same site 10 min prior to i.pl. Glu injection produced a significant increase in Glu-induced decreased PWTs compared with vehicle-pretreated controls. These data indicate that Glu induces tactile hypersensitivity in the L4 plantar dermatome, and this hypersensitivity is likely mediated by activation of NMDARs through intracellular PKA and PKC signaling pathways.

## 4. Discussion

### 4.1. Tactile Hypersensitivity Induced by the Antidromic ES of the Decentralized L5 SN

The efferent function of nociceptors can be achieved via the release of algesic substances from their peripheral terminals by noxious stimulation [2] and also by the antidromic propagation of impulses, for example, peripherally propagating injury discharge produced by damage to the peripheral nerve [28] and impulses traveling toward the periphery through the dorsal root reflex and axon reflex [2, 34]. The algesic substances released in the periphery via the activation of nociceptors are involved in the production of inflammation, which leads to nociceptor sensitization [1, 8]. Thus, it is possible that algesic substances released from the peripheral terminals of nociceptors may sensitize nearby nociceptors through a diffusion or reflex action. However, there has been much controversy over whether the sensitization of nociceptors spreads beyond the area of skin innervated by the injured or stimulated nerve [35–41]. This controversy is due, in part, to the use of different methods for nociceptor stimulation and different animal species for performing the experiments. Thus, conclusions have been drawn from conflicting and contradictory data.

The tetanic ES of decentralized L5 SN is a useful way to examine the involvement of peripherally propagating impulses and subsequently released algesic substances in pain hypersensitivity without considering the involvement of central sensitization by employing L5 DR-bearing rats, in which the central access of nerve impulses evoked by the ES of L5 SN is completely blocked. In the present study, the finding that tactile hypersensitivity developed in the center of the circle surrounded by the tori of the hind-paw (i.e., the midpoint of the L4 plantar dermatome) following the ES of the decentralized L5 SN indicates that skin hypersensitivity can occur near but outside the area of skin innervated by the stimulated L5 SN (Figures 1 and 2). Although neural mechanism of tissue hypersensitivity beyond the area of original injury, that is, secondary hyperalgesia, is generally considered to result from the sensitization of spinal dorsal horn neurons, a solid conclusion has not yet been drawn [42, 43]. The behavioral hypersensitivity observed in the present study can be accounted for by the sensitization of nociceptors in the L4 plantar dermatome rather than central mechanisms. The importance of nociceptors supplying the L4 dermatome in retaining behavioral hypersensitivity seen in our model rats has previously been revealed by the complete blockage of such hypersensitivity after the elimination of C-nociceptors in the L4 SN with local capsaicin treatment [28].

One might argue that a prior L5 DR can alter the properties of central neurons to affect the tactile sensitivity in the L4 dermatome. The barrage of impulses elicited briefly at the time of the dorsal root transection, degeneration of proximal stump of transected dorsal root, and enlarged receptive fields of dorsal horn neurons in the L5 spinal segment might be involved. Indeed, a mild tactile hypersensitivity was induced in the L4 dermatome by the L5 DR. Although the underlying mechanisms involved are not clear, this mild hypersensitivity following the L5 DR completely vanished in less than a week and did not reappear thereafter throughout the 12-week observation period [28], suggesting a return of response properties of affected neurons to the normal state before the ES experiment commenced. In the present study, observed plasma extravasation by tetanic ES of the L5 SN was not inducible by the L5 DR alone (data not shown), suggesting no noticeable effects of a prior L5 DR on response properties of nociceptors in the periphery. Thus, it is possible to assume that the tactile hypersensitivity in the L4 dermatome following the ES of the decentralized L5 SN and subsequent pharmacological modulation would not be confounded by a prior L5 DR.

### 4.2. Importance of NMDARs in Antidromic ES-Induced Hypersensitivity

The importance of the peripheral Glu and its receptors in developing tactile hyperalgesia has been acknowledged. The peripheral inhibition or activation of specific GluRs has been shown to attenuate enhanced tactile sensitivity in various experimental animal models of pain, for example, inflammatory [17, 19, 21, 22] and neuropathic [15, 23] pain, which are believed to be mediated at least in part by the increased sensitivity of nociceptors to tactile stimuli [44, 45]. In the present study, the local blockade of NMDARs or group-I mGluRs or the local activation of group-II mGluRs in the L4 dermatome completely blocked the decentralized L5-SN-ES-induced tactile hypersensitivity (Figure 3), and an NMDAR antagonist administered locally into the L4 dermatome reversed such already established hypersensitivity (Figure 4). These findings suggest the expansion of the area of mechanically hypersensitive skin to the L4 dermatome, which can be accounted for by the spread of nociceptor sensitization through the diffusion of Glu into the L4 dermatome from the release site in the L5 dermatome.

It is well known that NMDARs play a key role in the central sensitization of spinal nociceptive neurons [46–48]. In addition, the data in the present study indicate the importance of peripheral NMDARs but not AMPA/kainate receptors in the induction and maintenance of behavioral hypersensitivity (Figures 3 and 4), in accordance with a previous report in which the local inhibition of peripheral NMDARs in humans prevented the development of secondary hyperalgesia by a peripheral action [43]. Our data also revealed that the behavioral hypersensitivity could be blocked by the activation of peripheral group-II mGluRs, which suggests a desensitization of peripheral nociceptors via the activation of group-II mGluRs. A previous study has shown that group-I mGluRs are coexpressed with group-II mGluRs in peripheral afferent neurons [49]. In addition, the intrathecal administration of group-I mGluR agonists produces hyperalgesia [50], and the activation of group-I mGluRs results in the hyperexcitability of spinal dorsal horn neurons [51]. Thus, the coactivation of group-I and -II mGluRs by Glu may produce no changes in the excitability of nociceptors and subsequent behavioral sensitivity. We have previously shown that the local administration of group-I mGluR antagonists and group-II mGluR agonists in the periphery each hinders the initiation of tactile hypersensitivity in our modified neuropathic pain model whereas a nonselective antagonist of mGluR produces no such effect on hyperalgesia [15]. Although the coactivation of group-I and group-II mGluRs by Glu is less likely to participate in nociceptor sensitization and behavioral hypersensitivity, the use of substances that selectively block group-I mGluRs or selectively activate group-II mGluRs together with those that block NMDARs may prevent pain hypersensitivity related to nociceptor sensitization in the periphery.

### 4.3. Involvement of PKA and PKC in Antidromic ES-Induced Hypersensitivity

Some signal transduction receptors essential for sensing noxious mechanical stimuli have been identified in sensory neurons, which include the transient receptor potential Vanilloid 1 and Ankyrin 1 (TRPV1 and TRPA1) and piezo-type mechanosensitive cation channels (Piezo). For example, TRPA1 which is sensitized through various intracellular kinases such as PKA and phospholipase C (PLC) by inflammatory mediators, including bradykinin and prostaglandins, plays a critical role in tactile hyperalgesia [52–55].

We demonstrated in the present study that antidromic nerve stimulation-induced tactile hypersensitivity was blocked by pretreatment with inhibitors of PKA and PKC in the periphery (Figure 5), which indicates the involvement of activated peripheral PKA and PKC in the initiation of tactile hypersensitivity. The tactile hypersensitivity observed in our study can be considered to be the result of the PKA- and PKC-dependent sensitization of nociceptors to tactile stimuli mediated by peripheral Glu. There are several ways to account for such nociceptor sensitization. First, peripheral Glu activates adjacent nociceptors to release neuropeptides that attract and activate immune cells to release inflammatory mediators, resulting in nociceptor sensitization through protein kinase-dependent activation of transduction receptors as described above. Indeed, there are numerous pieces of evidence implying that SP and CGRP released from the peripheral terminals of primary sensory neurons induce nociceptor sensitization through the stimulation of immune cells to release inflammatory mediators [56–61]. Piezo2 on sensory neurons also can be involved in mediating inflammatory mediator-induced nociceptor sensitization. The potentiation of the activity of piezo2 through PKA and PKC has been found to mediate bradykinin-induced tactile hyperalgesia [62, 63]. Second, peripheral Glu, which binds to NMDARs on the nearby nociceptors, can sensitize these nociceptors through the intracellular protein kinase-dependent activation of transduction receptors. It has been shown that NMDA-induced tactile hyperalgesia is mediated by the activation of TRPV1 through CaMKII and PKC signaling cascades in trigeminal ganglion neurons [26, 27]. Third, the potentiation of NMDAR activity by mGluRs in sensory neurons via intracellular signaling pathways can be involved in NMDAR-mediated sensitization of nociceptors. Several pieces of evidence support this possibility. The activation of group-I mGluRs facilitates NMDAR-mediated responses in spinal dorsal horn neurons in the inflammatory hyperalgesic state [51]. The enhanced nociceptive processing in amygdala neurons by the activation of group-I mGluRs is mediated by reactive oxygen species (ROS) that activate PKA and ERK [64, 65]. ROS are known to serve as important intracellular signaling molecules in peripheral and central pain mechanisms [66–69].

Our findings that tactile hypersensitivity induced by antidromic stimulation of L5 SN or by i.pl. Glu injected into the L4 dermatome was blocked by pretreatment with an NMDAR antagonist and both PKA and PKC inhibitors in the L4 dermatome (Figures 3, 5, and 7) strongly support the idea that peripherally released Glu sensitizes nearby nociceptors through PKA- and PKC-dependent mechanisms mediated by NMDAR activation to induce pain hypersensitivity. Although, in the present study, we have not attempted to determine specific neuronal cell types involved in peripheral Glu- or ES-induced pain hypersensitivity and PKA/PKC expression patterns, unmyelinated C-nociceptors are likely critically involved in this pathway. Most of all, capsaicin sensitive afferents are clearly required for ES-induced mechanical hypersensitivity [28]. Also, the fact that candidate molecules responsible for the Glu- or inflammation-induced mechanical hypersensitivity via the PKA or PKC signaling pathway (e.g., TRPV1 [26, 27], TRPA1 [54, 55], and TTX-resistant sodium channels [70, 71]) are preferentially expressed on C-nociceptors supports this notion. Nevertheless, further study is warranted to confirm this possibility.

The already established tactile hypersensitivity following antidromic nerve stimulation was partially reversed by the blockade of NMDARs but not AMPA/kainate receptors, group-I mGluR, PKA, or PKC (Figures 4 and 6), which suggests that hypersensitivity is partly maintained by NMDAR activation. These results raise the possibility of the involvement of other NMDAR-mediated signaling molecules, such as ROS, PLC, and ERK, various transduction receptors, and other types of mGluRs in the maintenance of this hypersensitivity.

## 5. Conclusions

Our data suggest that peripheral Glu released by the antidromic stimulation of the decentralized SN results in the expansion of the area of tactile hyperalgesia via the spread of nociceptor sensitization, which is mediated by the diffusion of Glu into the skin near the release site. Our data also imply that intracellular PKA- and PKC-dependent mechanisms mediated mainly by NMDAR activation are involved in Glu-induced nociceptor sensitization and subsequent tactile hypersensitivity.
